# ERK-dependent phosphorylation of the linker and substrate-binding domain of HSP70 increases folding activity and cell proliferation

**DOI:** 10.1038/s12276-019-0317-0

**Published:** 2019-09-26

**Authors:** Semi Lim, Dae Gyu Kim, Sunghoon Kim

**Affiliations:** 1 Medicinal Bioconvergence Research Center, College of Pharmacy, Seoul National University, Seoul, Korea; 2 Department of Molecular Medicine and Biopharmaceutical Sciences, Graduate School of Convergence Science and Technology, College of Pharmacy, Seoul National University, Seoul, Korea

**Keywords:** Phosphorylation, Chaperones

## Abstract

The enhanced productive folding of translated polypeptides by heat shock protein 70 (HSP70) is often required for the survival of cancer cells. Although the folding activity of HSP70 is considered a significant determinant of the progression of cancer cells, it is still unknown how this activity could be regulated. Here, we report that the phosphorylation of HSP70 facilitates its folding activity, enhancing cell proliferation. Mass spectrometry identified the serine residues at positions 385 and 400 in the linker and substrate-binding domains of HSP70, respectively, as sites of phosphorylation mediated by EGF signaling, and this result was further confirmed by site-directed mutagenesis. ERK is known to be a specific kinase. The phosphorylation of the two sites induces the extended conformation of HSP70 via the regulation of the binding of the linker to the nucleotide- and substrate-binding domains, augmenting the binding affinity of HSP70 to substrates and enhancing its folding activity; this ultimately results in pro-proliferative effects. Cell lines harboring activated ERK showed increased phosphorylation of HSP70, and a positive correlation between the phosphorylation of HSP70 and the activity of ERK was observed. Thus, this study demonstrated that the ERK-dependent phosphorylation of HSP70 facilitated its folding activity and cellular proliferative function.

## Cancer: Unravelling controls of protein folding

When an enzyme adds phosphate groups to the protein HSP70 it enhances the role of this protein in the folding of other proteins, leading to increased cell proliferation. HSP70’s participation in the folding of other proteins can be crucial for the multiplication of cancer cells. Sunghoon Kim and colleagues at Seoul National University, South Korea, investigated factors controlling the activity of HSP70 in cultured human cells. They discovered that the enzymatic addition of phosphate groups to two specific regions of HSP70 increases its protein-folding activity, and hence accelerates cell proliferation. Molecular details of how the addition of phosphate groups achieves this effect were also uncovered. The enzyme responsible is itself stimulated by a key growth factor protein. Understanding the full chain of regulatory events could reveal new approaches to anti-cancer drugs.

## Introduction

The 70-kDa heat shock protein (HSP70) is a member of a family of ubiquitous molecular chaperone proteins that play an important role in protein folding and protecting the cell from heat shock and toxic stresses^1,2^. In addition to its canonical function, HSP70 is critical for cell proliferation, apoptosis, and protein degradation in various diseases, especially cancer, and thus, it has been considered a potential target for anticancer therapeutics^3–5^. HSP70 is composed of two highly conserved domains, the nucleotide-binding domain (NBD) and the substrate-binding domain (SBD), which are connected via a flexible linker^6^. SBD is further divided into two parts: SBDβ, which is an existing substrate-binding pocket for binding with the client protein, and SBDα, also called the lid, which is involved in strengthening the binding to the client protein. For the proper folding of the client protein, HSP70 undergoes two conformational changes, which allow it to assume either an adenosine triphosphate (ATP)- or an adenosine diphosphate (ADP)-bound form^6^. When the client binds to the SBD of the ATP-bound form, HSP70 performs the hydrolysis of ATP, inducing a conformational change into the ADP-bound form, which closes and exposes the lid and linker^7,8^. After the release of the properly folded protein, HSP70 again transforms into the ATP-bound form, which reduces the linker dynamics^7,8^. In the past, only the fine-tuning of the nucleotide-dependent cycling of the HSP70 conformation has been studied as a determinant of protein folding; however, recently, a new aspect, posttranslational modification, has also attracted attention in the regulation of the function of HSP70.

Among the various posttranslational modifications, phosphorylation is the most transient; however, it is considered a strong determinant of various cellular signaling pathways^9^. The phosphorylation of HSP70 has been slightly elucidated in various cellular contexts by a few studies. During protein translation, the phosphorylation of HSP70 regulates its dimerization and protein degradation via the HSP70-binding protein CHIP^10,11^. The regulation of the cell cycle and apoptosis via the phosphorylation of HSP70 has already been reported, and the phosphorylation of HSP70 has been explored in terms of chemoresistance in cancer cells^12,13^. Studies of the phosphorylation of HSP70 have been focused on the phosphorylation of the NBD and SBD because of the lack of awareness of the significance of the linker, which is critical for regulating the activity of HSP70. However, several reports have recently focused on the fine-tuning of the dynamics of HSP70 via the linker^8,14^. Here, we report two phosphorylation sites, serine 385 and 400, that are located in the linker. Both phosphorylation sites are phosphorylated by ERK upon EGF signaling and induce the extended conformation of HSP70. The phosphorylation-dependent extended conformation leads to the enhanced activity of HSP70, resulting in increased cell proliferation.

## Materials and methods

### Cell culture and materials

293T and WI-26 cells were cultured in DMEM supplemented with 10% FBS and 1% penicillin/streptomycin in 5% CO_2_ at 37 °C. WI-38, A549, H460, HCC827, and H1650 cells were cultivated in RPMI as described above. All of the cells used and GFP-tagged ERK were gifted from the Biocon biobank (Seoul National University, Seoul, Korea). GFP-HSP70 (#15215) was purchased from Addgene and subcloned into the EcoRI/XhoI sites of pEXPR-IBA105. Each of the HSP70 domains was cloned into the EcoRI/XhoI sites of pEGFP-C3, pEGFP-N1 and pGEX4T-1. The point mutagenesis of HSP70 was performed with a QuikChangeII kit (Promega) according to the manufacturer’s instructions. EGF was purchased from Peprotech. MG-132 and antibody against p-Ser were purchased from Sigma. Antibodies against p-Thr, p-Tyr, p-ERK, and ERK were purchased from Cell Signaling Technology, and other antibodies were purchased from Santa Cruz Biotechnology. StrepMAB-classic-HRP for detecting Strep was purchased from IBA Lifesciences. U0126 (#1144, Tocris), SP600125 (#1496, Tocris), SB203580 (#8307, Sigma) and Fludarabine (#F9813, Sigma) were purchased from the respective suppliers. LY294002 (#440202) was purchased from MERK. siRNA against ERK was purchased from Invitrogen (#VHS40404 and #VHS40318).

### Identification of phosphorylation sites

Strep-HSP70-expressing 293T cells were incubated with or without EGF for 10 min and purified with a Strep-Tactin column. The purified HSP70 was analyzed by SDS-PAGE and subjected to in-gel digestion with trypsin/Lys-C (Promega). For the identification of phosphopeptides, the analysis of the peptide mixture was performed using a LTQ-Orbitrap Velos (Thermo Fisher Scientific) connected to an Easy-nano LC II system (Thermo Fisher Scientific) incorporating an autosampler. The data acquired in data-dependent mode to simultaneously record the full-scan mass and collision-induced dissociation (CID) spectra with multistage activation were analyzed. To identify the specific sites of phosphorylation in the phosphopeptides, the CID spectra were searched for peptides containing the modifications p-Ser, p-Thr and p-Tyr by a combination of database searches and the plotting of neutral loss chromatograms to show the characteristic loss of a phosphate group. The proteome Discoverer (version 1.3, Thermo Scientific, Waltham, MA USA) and Scaffold programs (version 4.8.4, Proteome Software Inc., Portland, OR) were used to validate the MS/MS-based peptide and protein identifications and to perform the quantitative analysis. The precursor mass tolerance and fragment mass tolerance were set to 25 ppm and 0.8 Da, respectively.

### **In vitrokinase assay**

The phosphorylation of HSP70 by active ERK1 protein (Enzo Life Sciences) was monitored using a Universal Kinase Activity Kit (R&D Systems). Then, 0.15 mM purified HSP70 and 1 mM ATP were mixed with 2–50 ng/ml ERK1 in phosphatase buffer containing 250 mM HEPES, 1.5 M NaCl, 100 mM MgCl_2_, and 100 mM CaCl_2_. The coupling phosphatase CD39L2 was added to generate inorganic phosphate from ADP resulting from the kinase reaction. After incubation at room temperature for 10 min, malachite green reagent was added to terminate the reaction. The amount of phosphorylated HSP70 was determined by measuring the OD at 600 nm in a microplate reader, and the experiments were repeated three times independently.

### Refolding assay

The Glow-Substrate peptide was unfolded by heating at 40 °C for 7 min and subjected to a refolding assay using the HSP70/HSP40 Glow-Fold Protein Refolding kit (BostonBiochem) according to the manufacturer’s instructions. For the quantification of the refolded amounts of peptide, the luciferase signal was measured by a luciferase reader (GloMax, Promega) at various reaction times (10, 20, 30, 40, 50, and 60 min). The experiments were independently repeated three times.

### Limited digestion assay

For the limited digestion assay, purified wild type HSP70 and the S385D/S400D mutants were incubated with proteinase K according to a previously reported method^15,16^. Ten micrograms of HSP70 protein was preincubated with 200 μM ATP at room temperature, and proteinase K was added in a dose-dependent manner. The enzymatic reaction was performed at room temperature and stopped by adding SDS sample buffer and boiling it at 100 °C for 10 min prior SDS-PAGE with Coomassie staining.

### Luciferase assay

Wild type HSP70 or the S385D/S400D or S385A/S400A mutants was cloned into pBiT1.1-N[TK/LgBiT], and the SmBiT fragment of pBiT2.1-N[TK/SmBiT] was cloned after the C-terminus of HSP70 to generate a construct in which LgBiT and SmBiT from nanoluciferase were attached to the N- and C-terminus, respectively, of HSP70. CHO cells ectopically expressing each plasmid were treated with EGF (50 μg/ml) for 30, 60, 90 or 120 min. After incubation for the indicated time, the luciferase activity was determined with the nanoluciferase assay system according to the manufacturer’s protocol (Promega). The experiments were independently repeated three times.

### Enzyme-linked immunosorbent assay

A total of 150 ng of purified NRLLLTG peptide was diluted in 0.05 M sodium carbonate buffer (pH 9.6) and coated onto a flat-bottom 96-well plate (Thermo). After overnight incubation, the plate was washed with PBST 3 times and blocked with PBST containing 1% BSA. Each well was washed with PBST, and wild type HSP70 or S385D/S400D mutant protein was added in a dose-dependent manner. After washing with PBST, the primary antibody against HSP70 and the secondary antibody were incubated sequentially. 3,3′,5,5′-tetramethylbenzidine (TMB) solution (Thermo) was used to detect the bound HSP70, and 1 N H_2_SO_4_ was used for developing and stopping the reaction. The absorbance was measured at 450 nm with a 620 nm reference, and all experiments were repeated three times.

### In vitro pull-down assay

Purified HSP70 was mixed with anti-HSP70 antibody and agarose G for 4 h and washed three times with 50 mM Tris-HCl (pH 7.4) binding buffer containing 100 mM NaCl, 0.5% Triton X-100, 10% glycerol, 1 mM EDTA, and protease inhibitor (Calbiochem) at 4 °C. The antibody-conjugated HSP70 proteins were incubated with ERK proteins. After 12 h, the coprecipitates with HSP70 were harvested by centrifugation at 2000 rpm and washed three times with binding buffer at 4 °C. The ERK and HSP70 in precipitates were detected by SDS-PAGE and immunoblotting using specific antibodies. The purified GST-tagged proteins were mixed with cell lysates containing GFP-tagged proteins in 50 mM Tris-HCl (pH 7.4) binding buffer containing 100 mM NaCl, 0.5% Triton X-100, 10% glycerol, 1 mM EDTA, and protease inhibitor (Calbiochem) at 4 °C. After 12 h, the GST-tagged proteins were precipitated by glutathione-Sepharose beads and washed with binding buffer three times, and the coprecipitates with GST proteins were separated by SDS-PAGE. The amounts of GFP- and GST-tagged proteins were detected by immunoblotting using anti-GFP antibody and Coomassie staining, respectively.

### Immunoprecipitation

The cells were lysed with 50 mM Tris-HCl (pH 7.4) lysis buffer containing 100 mM NaCl, 0.5% Triton X-100, 0.1% SDS, 10% glycerol, 1 mM EDTA, and protease inhibitor (Calbiochem). Whole cell lysates were mixed with an antibody against the protein of interest and preincubated with agarose G for 12 h. After incubation, the coprecipitates with agarose G were washed with cold lysis buffer without 0.1% SDS three times. The strep-tagged HSP70 was precipitated with a Strep-Tactin column (IBA) according to the manufacturer’s instructions. The coprecipitates with the bait proteins were subjected to SDS-PAGE and immunoblotting.

### Soluble and insoluble protein fractionation

The 293T cells were dose-dependently transfected with Strep-HSP70 wild type, S385A/S400A and S385D/S400D proteins and lysed with 50 mM Tris-HCl (pH 7.4) buffer containing 0.5% Triton X-100 and protease inhibitor (Calbiochem). After lysis for 30 min at 4 °C, the whole cell lysates were separated into supernatant and pellet fractions by centrifugation at 13,200 rpm for 15 min at 4 °C. The pellet fraction was dissolved in lysis buffer containing 1% SDS. The amounts of proteins in each fraction were measured via spectrophotometry using Bradford solution (Bio-Rad). The experiments were independently repeated three times.

### Xenograft assay

GFP-tagged HSP70 wild type (WT), S385A/S400A or S385D/S400D protein was introduced into H460 cells, and the cells were cultured in medium containing 800 μg/ml G418 (Duchefa) for two weeks. After selection, the settled colonies were picked, and the level of expressed HSP70 was validated by immunoblotting using a specific antibody against GFP (Santa Cruz Biotechnology). The stably expressing cells (1 × 10^7^) were subcutaneously inoculated into the left and right sites of the backs of 7-week-old female BALB/cSLC-*nu/nu* mice (Central Lab. Animal Inc., Korea) (*n* = 3/group), and tumor progression was monitored for two weeks. The embedded tumor volumes and body weights were measured three times a week during the experimental period. The weights of the embedded tumors were measured after sacrifice. The animal experiments were conducted in compliance with the University Animal Care and Use Committee guidelines at Seoul National University.

### Anchorage-independent colony formation assay

Strep-HSP70 wild type or S385A/S400A or S385D/S400D mutant protein was introduced into 293T cells. The cells were subjected to an anchorage-independent colony formation assay using a cell transformation assay kit (Cell Biolabs, Inc.) according to the manufacturer’s instructions. After cell culture for 1 week, the soft agar was solubilized, and the colonies were stained with MTT solution (Sigma) and counted. The experiments were independently repeated three times.

### Cell viability assay

293T cells (1 × 10^4^) ectopically expressing Strep-HSP70 wild type protein or the S385A/S400A or S385D/S400D mutants were cultured in a single well of a 96-well plate for 24 h. The cells in each well were treated with 10 μl of MTT solution (5 mg/ml, Sigma) and incubated for 1 h at 37 °C. After discarding the culture media, the precipitated formazan crystals were dissolved in 100 μl DMSO (Duchefa). The absorbance was measured at 560 nm using a microplate reader (Sunrise, TECAN). The experiments were independently repeated three times.

### Cell toxicity assay

293T cells expressing wild-type Strep-HSP70, S385A/S400A or S385D/S400D protein were seeded in 96-well white plates and cultured overnight. The cells were treated with different concentrations of VER155008 (2.5, 5, 10, and 20 μM) for 6 h, and the dead cells were evaluated with the CytoTox-Glo kit (Promega). After adding a mixture of AAF-Glo substrate and buffer, the cells were incubated at 37 °C for 10 min, and the luminescence signal from the dead cells was measured by a luciferase reader (GloMax, Promega). The experiments were independently repeated three times.

### Quantification and statistical analysis

The statistical tests were performed with Prism (GraphPad). A value of *P* < 0.05 was considered statistically significant. All error bars represent the standard deviation (s.d.). For the quantitative data, the statistical parameters are reported in the figure legends.

## Results

### ERK phosphorylates HSP70

**Fig. 1:**
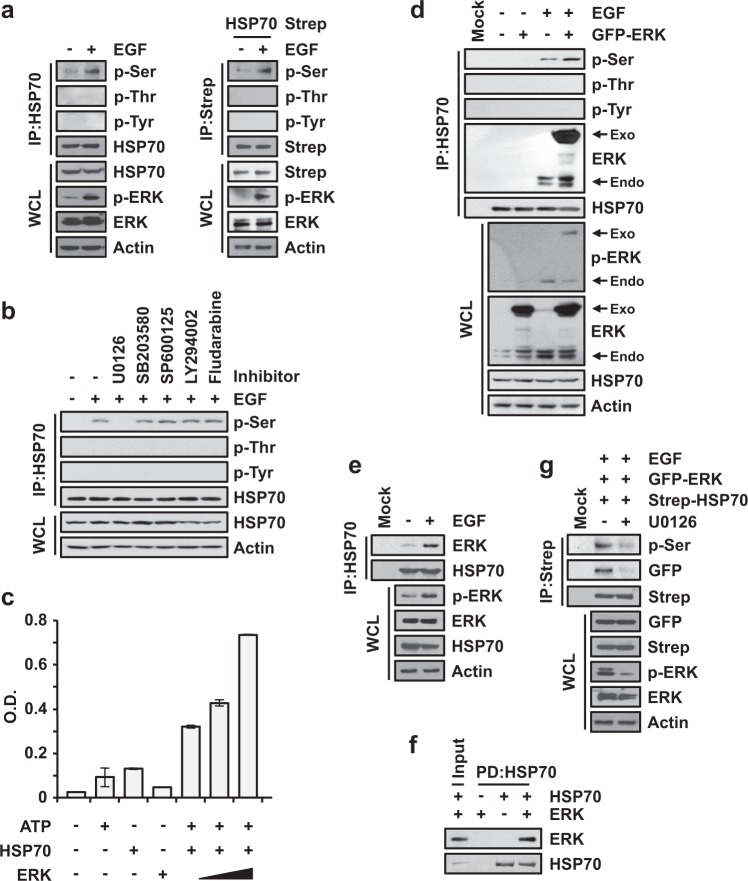
EGF-dependent phosphorylation of HSP70 via ERK. **a** Determination of the phosphorylation of residues in HSP70 mediated by EGF stimuli. After starvation, H460 cells were treated with EGF for 10 min, and endogenous HSP70 was precipitated by anti-HSP70 antibody (Left). 293T cells expressing Strep-HSP70 were incubated in medium containing EGF as described above, and HSP70 was precipitated using streptavidin-Sepharose beads (Right). Precipitates were subjected to SDS-PAGE and immunoblotting using the indicated antibodies. p-ERK and actin were used as a positive control for EGF signaling and as a loading control, respectively. **b** Identification of the kinase responsible for the phosphorylation of HSP70 upon EGF stimulation. 293T cells were preincubated with the indicated inhibitors and treated with EGF for 1 h and for 10 min. Phosphorylation was detected as described above. U0126 (1 μM), SB203580 (1 μM), SP600125 (10 μM), LY294002 (10 μM), and fludarabine (1 μM) were used as inhibitors for targeting the MAPK, PI3K-AKT, and JAK-STAT pathways in EGF signaling. **c** Determination of HSP70 phosphorylation in vitro. The indicated combinations of ATP, HSP70, and ERK were used for the in vitro phosphorylation assay. The experiments were independently repeated three times, with the error bars denoting the S.D. **d** Starved 293T cells containing GFP-ERK were treated with EGF for 10 min as indicated and subjected to immunoprecipitation as described above. Exogenous and endogenous ERK were determined by immunoblotting using anti-GFP and anti-ERK specific antibodies, respectively. **e** EGF-dependent endogenous binding between HSP70 and ERK. Starved H460 cells were treated with or without EGF for 10 min and subjected to immunoprecipitation with an antibody against HSP70. Coprecipitation of ERK with HSP70 was observed using an antibody against ERK. **f** In vitro binding between HSP70 and ERK. Purified HSP70 and ERK proteins were mixed, and HSP70 was precipitated by an anti-HSP70 antibody. ERK proteins coprecipitated with HSP70 were identified by SDS-PAGE and immunoblotting using an antibody against ERK. **g** Starved 293T cells containing GFP-ERK and Strep-HSP70 were treated with U0126 and EGF for 1 h and for 10 min, respectively, as indicated. The cells were subjected to immunoprecipitation as described above

The enhancement of the productive folding of translated peptides is very important for the enhanced proliferation of cancer cells^4^. Although the levels of heat shock proteins (HSPs) in cancer cells are not associated with successful folding, translated peptides are folded well^17,18^. Since the activity of HSP70 in cancer cells has been reported to be regulated by phosphorylation, and HSP70 is a prerequisite for proper folding via other chaperone proteins in the chaperone network^3,19,20^, we investigated whether HSP70 is phosphorylated via the EGF signaling pathway, which is critical for cancer progression^21,22^. By using the immunoprecipitation of H460 cells, +EGF signaling-mediated phosphorylation of serine, but not threonine and tyrosine, residues in endogenous HSP70 was monitored (Fig. 1a, left), and the same results were observed for overexpressed HSP70 (Fig. 1a, right). To determine the specificity of the signal, we also tested whether the phosphorylation of HSP70 was induced by other signals. Upon the stimulation of TNF-α and TGF-β, which are significant signaling molecules involved in cancer^23,24^, no phosphorylation of HSP70 was observed, but phosphorylation was observed as a result of EGF signaling (Supplementary Fig. 1a, left). Additionally, we monitored the phosphorylation of HSP90, another abundant HSP, under the same conditions as above, and no phosphorylation of HSP90 was observed (Supplementary Fig. 1a, right). To determine the kinase responsible for the phosphorylation of HSP70, we treated cells with several inhibitors, including U0126, SB203580, SP600125, LY294002 and fludarabine, to target the downstream kinases of the EGF signaling pathway^25^. EGF-dependent phosphorylation of serine residues in HSP70 was diminished only by treatment with U0126, an inhibitor of the mitogen-activated protein kinase kinases MEK-1 and MEK-2, implying that ERK is a specific kinase involved in the phosphorylation of HSP70 via EGF signaling (Fig. 1b). To investigate whether ERK directly phosphorylates HSP70, we performed an in vitro kinase assay using purified ERK proteins and confirmed the ERK-mediated increase in HSP70 phosphorylation in a dose-dependent manner (Fig. 1c), suggesting that ERK is a kinase directly involved in the phosphorylation of HSP70. The phosphorylation of serine residues in HSP70 was also observed during the overexpression of ERK upon EGF signaling (Fig. 1d), implying that the EGF signaling-mediated activation of ERK is essential for the phosphorylation of HSP70. To further validate the significance of ERK as a kinase, we also checked the phosphorylation of HSP70 when ERK was knocked down, which revealed the decreased phosphorylation of endogenous HSP70 (Supplementary Fig. 1b) and suggested that ERK is a kinase specific to the EGF-dependent phosphorylation of HSP70.

Next, we investigated whether the binding between HSP70 and ERK is induced via EGF signaling, as binding between substrate and kinase is essential for phosphorylation. When cells were treated with EGF, endogenous and exogenous binding of ERK to HSP70 was induced (Fig. 1d and e, and Supplementary Fig. 1c), suggesting that the EGF-dependent phosphorylation of HSP70 is mediated by ERK. The direct interaction of the two proteins was further confirmed by an in vitro pull-down assay using purified ERK or an extract expressing ERK with purified HSP70 (Fig. 1f and Supplementary Fig. 1d). We also checked whether the EGF signaling-mediated activation of ERK was required for the binding of the two proteins. Upon treatment with U0126, the binding of ERK to HSP70 and the level of HSP70 phosphorylation declined, suggesting the significance of ERK activity for binding to HSP70 (Fig. 1g). Taken together, ERK was revealed as the specific kinase responsible for EGF signaling-dependent phosphorylation of HSP70.

### Serines at positions 385 and 400 in HSP70 are phosphorylated by EGF stimuli

**Fig. 2:**
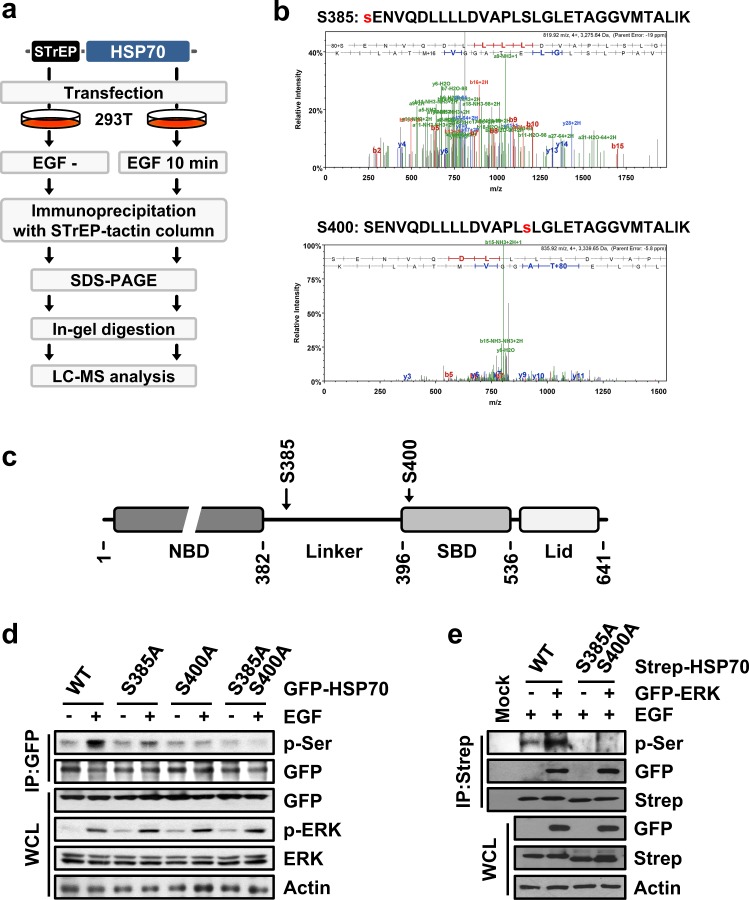
Identification of HSP70 phosphorylation residues. **a**, **b** LC-MS analysis for the identification of the phosphorylated residues of HSP70 **a** Strategy used for the identification of HSP70 phosphorylation sites via LC-MS analysis. **b** Mass spectrum indicating the phosphorylation of HSP70 serine residues at positions 385 (upper) and 400 (bottom). The identified peptide sequence, including the phosphorylated residue, is presented above its spectrum. Phosphorylated serines are colored red. **c** Cartoon showing HSP70 domain arrangement. NBD and SBD indicate the nucleotide-binding domain and the substrate-binding domain, respectively. Serine residues at positions 385 and 400 were located in the linker and SBD regions, respectively. **d** The indicated mutants-expressing 293T cells were either treated or not treated with EGF, and phosphorylation was determined by immunoblotting using specific antibodies. **e** Strep-HSP70 and GFP-ERK were introduced into 293T cells as indicated. HSP70 phosphorylation and binding to ERK were detected by Western blotting

Next, we determined the phosphorylation sites of HSP70. Strep-HSP70-expressing 293T cells were treated with or without EGF, and the precipitated Strep-HSP70 was subjected to LC-MS analysis to identify the phosphorylated residues of HSP70 (Fig. 2a). Two other peptides containing phosphorylated serine residues at positions 385 and 400 were identified by mass spectrometry (Fig. 2b). In the structure of HSP70, serine 385 and 400 are located in the linker and N-terminus of the substrate-binding domain (SBD), respectively (Fig. 2c). To confirm that the residues identified via mass spectrometry were the actual phosphorylation sites, we generated the alanine substitution mutants S385A, S400A and S385A/S400A. 293T cells expressing each of these mutants were stimulated by treatment with EGF, and the phosphorylation of HSP70 was examined by immunoblotting. S385A- or S400A-expressing cells showed the mild induction of phosphorylation upon EGF treatment; however, we could not detect the induced phosphorylation of the double alanine mutant S385A/S400A, implying that serine 385 and 400 were the actual EGF signaling-dependent phosphorylation sites (Fig. 2d). Next, we further confirmed that these two residues are phosphorylated by ERK. The ectopic expression of ERK caused the phosphorylation of wild type (WT) HSP70 but not the HSP70 S385A/S400A double alanine mutant upon treatment with EGF (Fig. 2e). Therefore, the serine residues at positions 385 and 400 of HSP70 are phosphorylated by ERK.

### HSP70 phosphorylation enhances protein folding

**Fig. 3:**
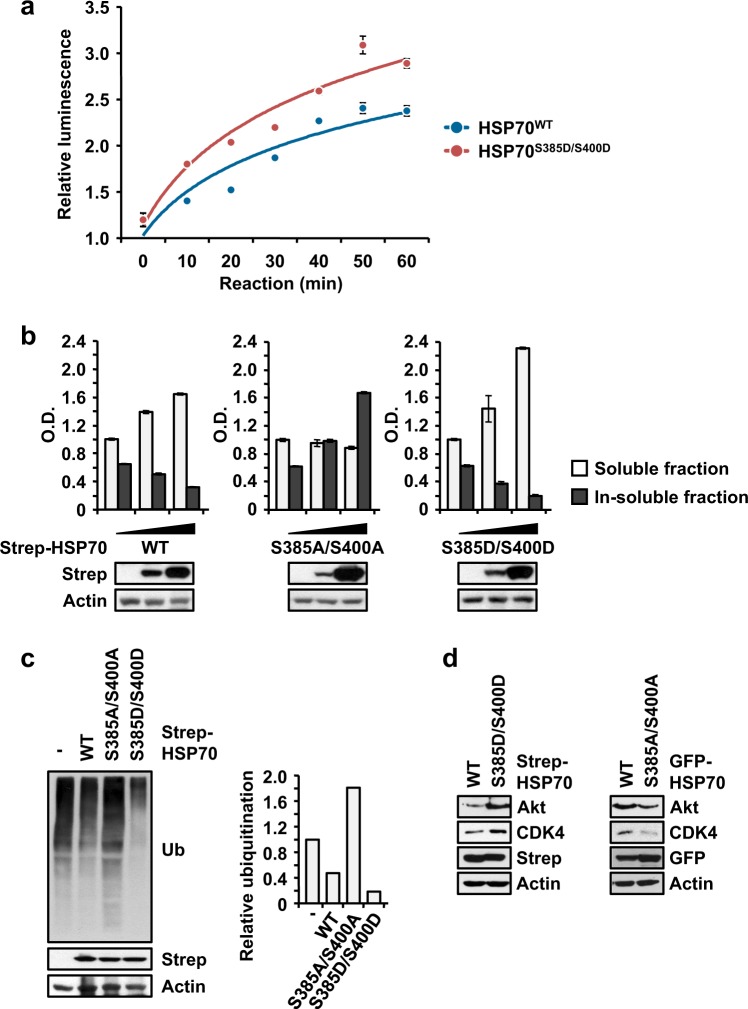
Significance of HSP70 phosphorylation for protein folding. **a** Refolding activity of phosphorylated HSP70. Purified wild type (WT) and double phospho-mimetic S385D/S400D HSP70 proteins were subjected to a refolding assay. The experiments were independently repeated three times, with the error bars denoting the S.D. **b** Determination of soluble and insoluble protein fractions following the phosphorylation of HSP70. Strep-HSP70 wild type (WT) and S385A/S400A and S385D/S400D mutant proteins were expressed in 293T cells. The soluble and insoluble protein fractions from whole cell lysates were separated and subjected to SDS-PAGE and immunoblotting using an anti-Strep antibody. Actin was used as a loading control. **c** Determination of ubiquitinated proteins via the phosphorylation of HSP70. The same cells as described above were treated with MG-132. The amounts of ubiquitinated proteins were determined by immunoblotting using a specific antibody against ubiquitin (Ub). The quantitated degree of ubiquitination is shown in the bar graph on the right. **d** The indicated HSP70 proteins were introduced into 293T cells, and the endogenous levels of Akt and CDK4 were evaluated by Western blotting using specific antibodies

To elucidate the significance of phosphorylation in cell proliferation, we first examined the canonical functioning and folding activity of HSP70. According to a refolding assay using purified wild type (WT) HSP70 and a double phospho-mimetic mutant, S385D/S400D, we observed that the S385D/S400D mutant showed ~1.5-fold higher folding activity compared to the WT (Fig. 3a). Next, we assayed the proteins that aggregated due to improper folding by monitoring the insoluble fractions of cell lysates. Strep-HSP70 WT, S385A/S400A or S385D/S400D was introduced into cells, and the soluble and insoluble fractions in total cell lysates were monitored. When Strep-HSP70 was expressed, the soluble protein fraction was increased, but the insoluble protein fraction was decreased (Fig. 3b, left). However, S385A/S400A- and S385D/S400D-expressing cells showed significant increases in the insoluble and soluble protein fractions, respectively (Fig. 3b, middle and right, respectively). These results suggested that the phosphorylation of HSP70 enhances its folding activity, resulting in the productive folding of client proteins. We also monitored the ubiquitination of whole proteins as a determinant of degradation due to the improper folding of proteins that were ectopically expressed by HSP70 WT or mutant cells. The cells were treated with a proteasome inhibitor, and the level of ubiquitination in total cell lysates was determined. The ectopic expression of WT HSP70 decreased the level of ubiquitination (Fig. 3c, second lane). However, cells expressing the HSP70 S385A/S400A and S385D/S400D mutants exhibited higher and lower levels of ubiquitination, respectively (Fig. 3c, third and fourth lane, respectively). These findings indicated the significance of HSP70 phosphorylation for its folding activity. We further explored the levels of the well-known client proteins Akt and CDK4 under different conditions of HSP70 phosphorylation. The expression of the double phospho-mimetic mutant S385D/S400D but not that of S385A/S400A led to an increase in the levels of these client proteins, as expected (Fig. 3d). To determine whether phosphorylated HSP70 directly affected the ubiquitination of clients, we also determined the ubiquitination of Akt and CDK4. The increased and decreased ubiquitination of these two clients was observed during the expression of S385A/S400A and S385D/S400D, respectively (Supplementary Fig. 2a), which was similar to the levels observed for the clients in Fig. 3d. Taken together, these results suggest that phosphorylation enhances the folding activity of HSP70, resulting in improved quality control and the proper folding of client proteins.

### Conformational change of HSP70 via phosphorylation

**Fig. 4:**
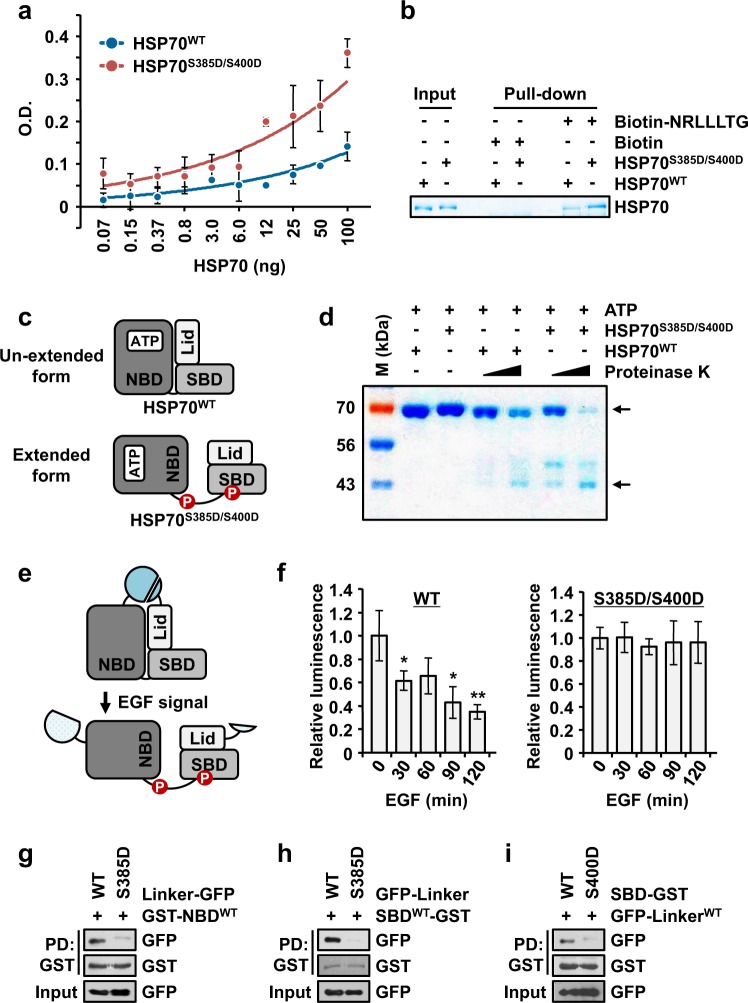
Phosphorylation-mediated conformational change of HSP70. **a** Enzyme-linked immunosorbent assay (ELISA) was used for monitoring the interaction between HSP70 and the NRLLLTG peptide. The binding affinity of the NRLLLTG peptide to wild type (WT) or double phospho-mimetic S385D/S400D HSP70 proteins was evaluated by ELISA. The experiments were independently repeated three times, with the error bars denoting the S.D. **b** In vitro pull-down assay showing the difference in the binding of NRLLLTG to the WT and S385D/S400D HSP70 proteins. Biotin-NRLLLTG or biotin was mixed with the two proteins and pulled down by streptavidin-Sepharose beads. The precipitates were separated by SDS-PAGE and subjected to Coomassie staining. **c** Cartoon showing the two different conformations of HSP70. **d** Limited digestion assay to confirm the conformation of HSP70. WT or S385D/S400D HSP70 was preincubated with ATP and mixed with varying concentrations of proteinase K. The digested proteins were separated and detected by SDS-PAGE and Coomassie staining. M denotes the size marker. The number on the left of the gel image denotes the molecular weight (kDa). **e**, **f** Determination of conformational change of HSP70 via a nanoluciferase assay. **e** Schematic diagram of the nanoluciferase assay used to check the EGF-dependent changes in HSP70 conformation. LgBiT and SmBiT from nanoluciferase were attached to the N- and C-termini, respectively, of HSP70 WT, S385D/S400D and S385A/S400A. In the presence of the EGF signal, the increased distance between the two fragments led to a decline in the luciferase signal. **f** The cells expressing each type of HSP70 were treated with EGF for varying times, and the luciferase signal was determined. The luciferase signals relative to that of the untreated sample are shown in the bar graph. The result for S385A/S400A is presented in Supplementary Fig. 3a. The experiments were independently repeated three times, with the error bars denoting the S.D. The statistical analysis was performed with Student’s two-tailed t-test (*P < 0.05, **P < 0.01). **g**–**i** Lysates containing the GFP-tagged S385D mutant of the HSP70 linker and the purified GST-HSP70-NBD (**g**) and -SBD (**h**) proteins were subjected to an in vitro pull-down assay. Purified HSP70-SBD protein with the S400D mutation was mixed with lysates expressing the GFP-tagged HSP70 linker for the in vitro pull-down assay (**i**)

To unveil how phosphorylation enhances the folding activity of HSP70, we first investigated the binding affinity of wild type HSP70 (WT) or the double phospho-mimetic mutant protein S385D/S400D for NRLLLTG, a well-known substrate peptide^26^. By using an enzyme-linked immunosorbent assay (ELISA), the binding affinities of the two proteins for NRLLLTG were calculated, and double phospho-mimetic HSP70 exhibited approximately 1.5 times higher affinity than WT HSP70 (Fig. 4a). A similar result was observed in the in vitro pull-down assay using NRLLLTG peptide and HSP70 proteins (Fig. 4b).

HSP70 has distinctly different unextended and extended conformations, and the flexible linker region in the extended form is more exposed than in the unextended form and possesses stronger binding affinity for the substrate^7^. Moreover, the addition of ATP leads to the transformation of the extended conformation into the unextended conformation^27^. Since phosphorylated HSP70 shows stronger binding affinity for the substrate and better folding activity than WT HSP70, we hypothesized that phosphorylated HSP70 shows an extended conformation and hence a more exposed linker and that the conformational change from the extended form to the unextended form is inhibited via phosphorylation, even after the addition of ATP (Fig. 4c). To prove this hypothesis, we conducted a limited digestion assay using proteinase K because the exposed linker region is easy to cleave with proteinase^15^. As shown in Fig. 4d, as expected, WT HSP70 was protected from digestion by proteinase K after the addition of ATP; however, ATP-dependent protection was lost in the case of the double phospho-mimetic HSP70, suggesting that phosphorylated HSP70 has an exposed linker region and a conformation similar to that of the ADP-bound form. However, there is a lack of understanding about the exact conformation of phosphorylated HSP70, so to further explore this issue, we constructed two fragmented subunits of nanoluciferase, LgBiT and SmBiT, which were used to tag the N- and C-termini of HSP70. This system emits a luciferase signal if the distance between these two fragments is decreased (Fig. 4e). The luciferase signal from WT HSP70 but not that from double phospho-mimetic or phospho-defective HSP70 declined upon exposure to EGF signaling in a time-dependent manner, (Fig. 4f and Supplementary Fig. 3a, respectively), suggesting that EGF signaling-mediated phosphorylation induces a conformational change from the unextended form to the extended form. Additionally, we further validated whether the EGF signaling-mediated activation of ERK affected the conformational change of HSP70. The luminescence signal from HSP70 upon stimulation with EGF was decreased, and additional ectopic expression of ERK in the presence of EGF led to a greater decrease (Supplementary Fig. 3b), implying that the EGF-mediated phosphorylation of HSP70 via ERK is required for the conformational change of HSP70 to occur. Thus, we concluded that the phosphorylation of serine residues 385 and 400 in HSP70 induced and enhanced the formation of the extended conformation, resulting in the stronger binding of HSP70 to its substrate.

To understand how each phosphorylated residue contributes to the extended conformation of HSP70, we analyzed the structure of HSP70 by determining the interaction of the linker and the SBD, which contain S385D and S400D, respectively (see Fig. 2c). Through a pull-down assay, it was revealed that the linker containing S385D failed to bind the NBD or SBD (Fig. 4g and h, respectively). In contrast, the SBD containing S400D also failed to bind the linker (Fig. 4i). These results indicated that each corresponding domain containing S385D or S400D failed to bind its adjacent interacting domain, implying that the phosphorylation of HSP70 leads to the extended conformation.

### Augmentation of cell proliferation via the phosphorylation of HSP70

**Fig. 5:**
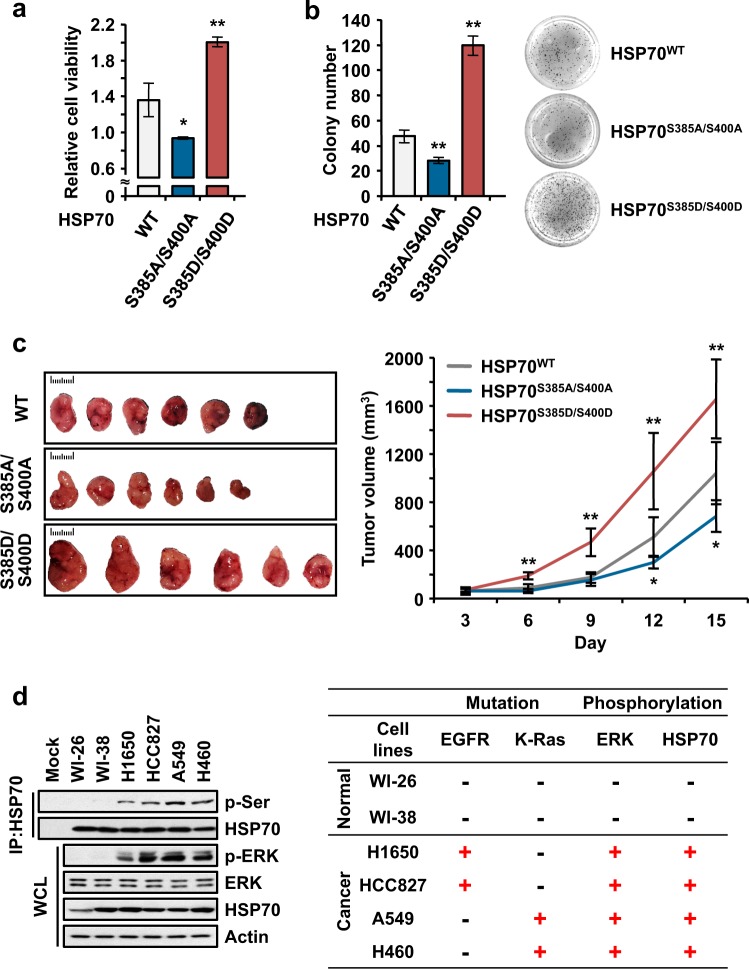
Significance of HSP70 phosphorylation to cell proliferation. **a**, **b** Determination of the cellular function of phosphorylated HSP70. **a** Wild type (WT) and S385A/S400A and S385D/S400D mutant HSP70 proteins were introduced into 293 T cells. The cells were subjected to an MTT assay to examine cell viability. **b** the Same cells were subjected to an anchorage-independent colony forming assay. The number of colonies was counted and is shown as a bar graph. The image on the right shows the settled colonies. All experiments were independently repeated three times, with the error bars denoting the S.D. The statistical analysis was performed with Student’s two-tailed t-test (*P < 0.05, **P < 0.01). **c** In vivo study showing phosphorylated HSP70-mediated tumor progression. H460 cells stably expressing WT HSP70 or the S385A/S400A or S385D/S400D mutants were subcutaneously xenografted into BALB/cSLC-*nu/nu* mice and monitored for two weeks. The tumor volume was monitored for the experimental period (right). Images of harvested tumors are shown on the left. The statistical analysis was performed with Student’s two-tailed *t*-test (*P < 0.05, **P < 0.01). **d** Cell line analysis showing the varying status of HSP70 phosphorylation. WI-26, WI-38, H1650, HCC827, A549, and H460 cells were subjected to immunoprecipitation to examine the phosphorylation of HSP70 (left). Mutations in EGFR and K-Ras and the phosphorylation of ERK and HSP70 are summarized in the table (right). Actin was used as a loading control

To investigate whether the enhancement of the folding activity of HSP70 via its phosphorylation leads to enhanced cell proliferation, we examined the viability of 293T cells ectopically expressing wild type (WT), S385A/S400A or S385D/S400D HSP70. First, we found that S385A/S400A- and S385D/S400D-expressing cells showed lower and higher cell viability than those expressing WT HSP70, respectively (Fig. 5a). Second, we also performed an anchorage-independent colony forming assay, which revealed a similar result to the cell viability assay, including the significant enhancement of colony formation in cells expressing S385D/S400D (Fig. 5b). Third, using a cytotoxicity assay, we found that double phospho-mimetic HSP70-expressing cells were protected from HSP70 inhibitor-mediated cell death. When cells expressing WT HSP70 were treated with VER155008 (VER), which inhibits HSP70 activity by interacting with the ATP-binding pocket in the NBD, cell death was enhanced owing to the inhibition of HSP70 activity (Supplementary Fig. 4a, gray). Similarly, S385A/S400A-expressing cells were more vulnerable to VER-mediated cell death than those expressing WT (Supplementary Fig. 4a, blue); however, VER could not induce cell death in S385D/S400D-expressing cells (Supplementary Fig. 4a, red), indicating that the induction of the extended conformation of HSP70 via phosphorylation might protect cells from VER-mediated cell death by augmenting HSP70 folding activity. Moreover, the findings revealed that the phosphorylation-mediated regulation of HSP70 activity was more important than ATP-mediated regulation. To confirm the direct effect on cancer progression of the phosphorylation of HSP70, we performed an in vivo study. We generated H460 cells stably expressing WT, S385A/S400A or S385D/S400D HSP70 and monitored the progression of embedded stable cells after xenograft assays. Similar to the above results, S385A/S400A- and S385D/S400D-expressing tumors showed declined and enhanced progression, respectively, compared with wild type tumors (Fig. 5c and Supplementary Fig. 4b). Altogether, enhanced activity via the phosphorylation of HSP70 resulted in enhanced cancer progression.

To further validate the pathological implications of HSP70 phosphorylation in cancer cells, we analyzed the basic phosphorylation of HSP70 in the absence of EGF signaling in normal and cancerous lung cells harboring mutations in EGFR or K-Ras, which is a well-studied activator of ERK^28^. Phosphorylated HSP70 was observed in the tested cancer cells but not in normal cells, indicating that cancer cells showing enhanced proliferation exhibited proper folding of the translated peptides due to the phosphorylation of HSP70, in addition to the other folding machinery (Fig. 5d, first lane on left). Interestingly, it was also observed that the phosphorylation of HSP70 was positively correlated with ERK activity, suggesting that ERK, as a kinase, mediates the phosphorylation of HSP70 (Fig. 5d, right). These results suggest that the activity of ERK is a critical determinant of HSP70 phosphorylation and that the promotion of protein synthesis was dependent on the activity of HSP70.

**Fig. 6:**
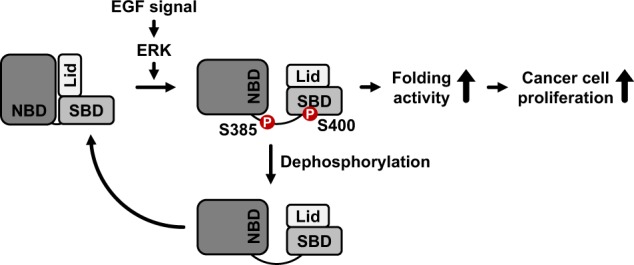
Schematic representation of HSP70 phosphorylation via ERK and its cellular function. Schematic model of the conformational change of HSP70 mediated by the phosphorylation of its serine residues at positions 385 and 400. In the presence of EGF signaling, the serine residues at positions 385 and 400 in HSP70 were phosphorylated by ERK. Phosphorylation enhances the extended conformation of HSP70, resulting in its augmented folding activity and the enhancement of tumor progression

Taken together, these results suggest a schematic model for the regulation of the folding activity of HSP70 via phosphorylation (Fig. 6). In the presence of EGF signaling, activated ERK phosphorylates the serine residues at position 385 in the linker region and at position 400 in the SBD of HSP70. Phosphorylated HSP70 assumes the extended conformation of HSP70, resulting in enhanced HSP70 folding activity and cancer progression promoted by cell proliferation. Through dephosphorylation, the extended conformation of HSP70 could be converted to the unextended conformation.

## Discussion

Cancer cells are characterized by rapid cell growth and division compared to normal cells, and rapid growth and division requires accurate and rapid protein synthesis^29^. Among the many processes of protein synthesis, accurate protein folding is critical to maintaining the exact structures of proteins, determining their function^3^. HSPs are chaperones that take charge of the proper folding of translated peptides^1^. Among HSPs, HSP70 is a well-studied and promising target for cancer therapy^4^. However, the level of HSP70 is not correlated with the amounts of the productively folded proteins in cancer cells; thus, protein folding efficiency has been studied^17^. This has raised the question of whether HSP70 is a real cancer target, and many studies have tried to answer this question. Here, we report for the first time that the phosphorylation of the linker region of HSP70 via ERK enhances protein folding activity, resulting in the rapid proliferation of cancer cells. We determined that serine residues 385 and 400 are phosphorylated by ERK upon EGF signaling and that phosphorylated HSP70 shows enhanced folding activity, enhancing cellular protein folding. We also determined the mode of action for the increase in folding activity via phosphorylation. The phosphorylation of serine residues at positions 385 and 400 in the linker and N-terminus of the SBD, respectively, leads to the induction of the extended conformation of HSP70, resulting in a strong binding affinity between substrate and HSP70.

HSP70 contains two domains, the NBD and the SBD, which are divided by a flexible linker region^6^. Upon ATP hydrolysis, HSP70 assumes two distinct conformations, unextended and extended, and each conformation shows varied folding activity^7^. Recently, the dynamics of the conformational change via the exposed flexible linker region have been studied^14^; however, no factor has been identified that affects the dynamics of the linker region. Our discovery regarding the phosphorylation of serine residue 385 in the linker region of HSP70 could explain the dynamics of the linker region. The phosphorylated serine 385 residue in the linker region failed to bind to the NBD and the SBD of HSP70. This result suggests that phosphorylation of the linker of HSP70 increased its exposure and enhances the dynamic conversion to the extended conformation. In addition, we identified the serine 400 residue as another phosphorylation site. Serine 400 is structurally near SBDα, which is the lid domain, in the N-terminus of SBD. Our results show that the phosphorylation of serine 400 in the SBD leads to a decrease in its binding with the linker region. This result suggests the possibility that the lid firmly covers the substrate-binding pocket of SBD, resulting in the strong binding affinity of the substrate to the SBD because the lid is closed in the extended form of HSP70^27^. Thus, the phosphorylation sites at serine 385 and 400 could be efficient targets to regulate the structural dynamics of HSP70.

Recently, a few research groups have reported on the phosphorylation sites in the NBD and the SBD of HSP70 and revealed the significance of phosphorylation in pathological conditions^30^. In cancer cells, hyperactive kinases phosphorylate HSP70, resulting in two hot spots for phosphorylation, one in the NBD and another in the SBD^31^. Increased HSP70 phosphorylation by hyperactive kinases promotes cancer progression via the regulation of protein stabilization, the cell cycle, and responses to cancer drug^10,12,13^. However, there have been no reports about phosphorylation in the linker of HSP70 because the importance of the linker for the dynamics and function of HSP70 has not been thoroughly studied. Here, we first reported the phosphorylation site at serine 385 in the linker region and its significance to the process of folding and cellular effects. Although we need to further elucidate which conformation is favorable to phosphorylation by ERK, the functional analysis of the phosphorylation of serine residue 385 in HSP70 in this report is crucial for understanding the regulation of the folding activity of HSP70 via phosphorylation in the linker region.

We also revealed that the control of HSP70 activity via phosphorylation ultimately affects cellular global ubiquitination. There are many HSPs involved protein folding, and several HSPs are involved in the efficient folding process of only a single client^1,32^. In addition to direct folding via HSP70, HSP70 is essential for the folding processes involving various other HSPs because HSP70 is a prerequisite for folding via other HSPs^3,19,20^. If HSP70 fails to function in the chaperone network, the subsequent sequential folding cascade cannot be successful. Therefore, the activation of HSP70 (S385D/S400D) via phosphorylation could lead to a decline in global ubiquitination compared with that of wild-type HSP70, in addition to a decline in direct folding efficacy via HSP70.

ERK is a well-studied kinase activated by growth signaling^28^. Activated ERK mainly regulates transcription factors associated with protein translation and translational machinery molecules, facilitating cell proliferation and survival^33^. In this study, we revealed that the activation of ERK via EGF signaling controls the conformation of HSP70 via phosphorylation, resulting in its augmented folding activity. Thus, a novel function of ERK, its direct regulation of productive folding, is revealed in this study.

HSPs have been considered attractive therapeutic targets for the treatment of cancer for a long time; however, targeting HSPs presents a significant challenge because of redundancy. In fact, the reason many trials targeting HSPs have failed was the generation of toxicity in normal cells in addition to cancer cells^5^. In this regard, phosphorylated HSP70 could be a very attractive target for eliminating cancer cells. The phosphorylation of HSP70 could be considered a pathological, not physiological, phenomenon, and thus, therapeutic agents used to target phosphorylated HSP70 could be specific to cancer cells rather than normal cells. Resistance and addiction to therapeutic agents targeting phosphorylated HSP70 is not likely to be observed either, since post-translational modification, especially phosphorylation, is transient. Since the phosphorylation of HSP70 is also tightly dependent on the activation of ERK, and many studies have reported the enhanced activation of ERK in cancer cells^34^, many patients could benefit from this approach. Thus, targeting phosphorylated HSP70 for treating cancer could be a new alternative strategy to solve existing problems.

## Supplementary information

Supplementary Figure 1–4

Supplementary figure legends
