# Supplementary material for: ERK-dependent phosphorylation of the linker and substrate-binding domain of HSP70 increases folding activity and cell proliferation
Source: Exp Mol Med. 2019 Sep 26;51(9):112. doi: 10.1038/s12276-019-0317-0 (PMC6802635; doi:10.1038/s12276-019-0317-0)
Supplement: Supplementary file 1 — Supplementary Figure 1–4 [file 12276_2019_317_MOESM1_ESM.pptx]

## Slide 1
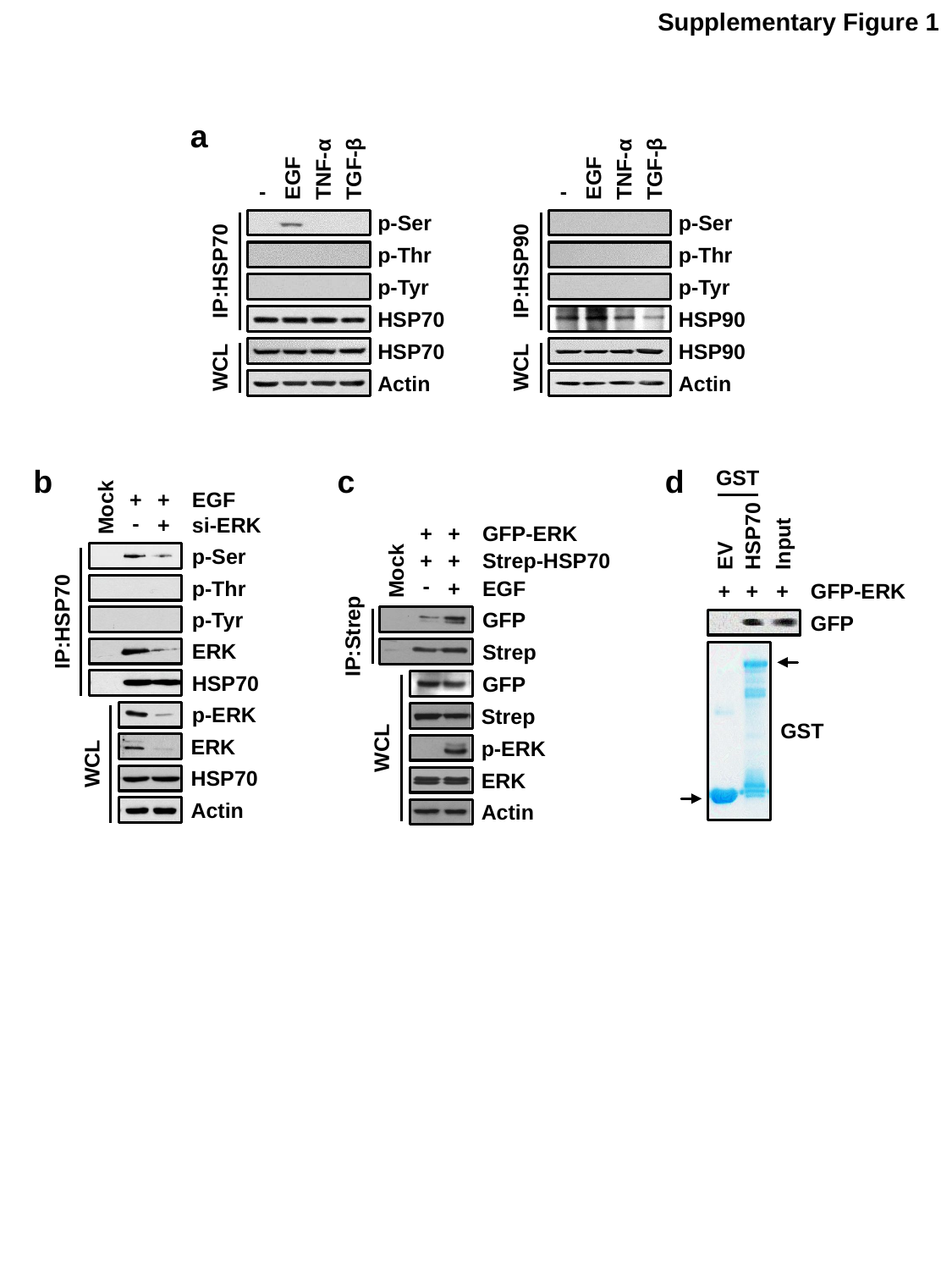

Supplementary Figure 1
EGF
TNF-α
TGF-β
 -
p-Ser
p-Thr
IP:HSP70
p-Tyr
HSP70
HSP70
WCL
Actin
EGF
TNF-α
TGF-β
 -
p-Ser
p-Thr
IP:HSP90
p-Tyr
HSP90
HSP90
WCL
Actin
a
Mock
+
+
EGF
-
+
si-ERK
p-Ser
p-Thr
p-Tyr
IP:HSP70
ERK
HSP70
p-ERK
ERK
WCL
HSP70
Actin
b
c
d
GST
Input
EV
HSP70
+
+
+
GFP-ERK
GFP
GST
+
+
GFP-ERK
+
+
Strep-HSP70
Mock
-
+
EGF
GFP
IP:Strep
Strep
GFP
Strep
p-ERK
WCL
ERK
Actin

## Slide 2
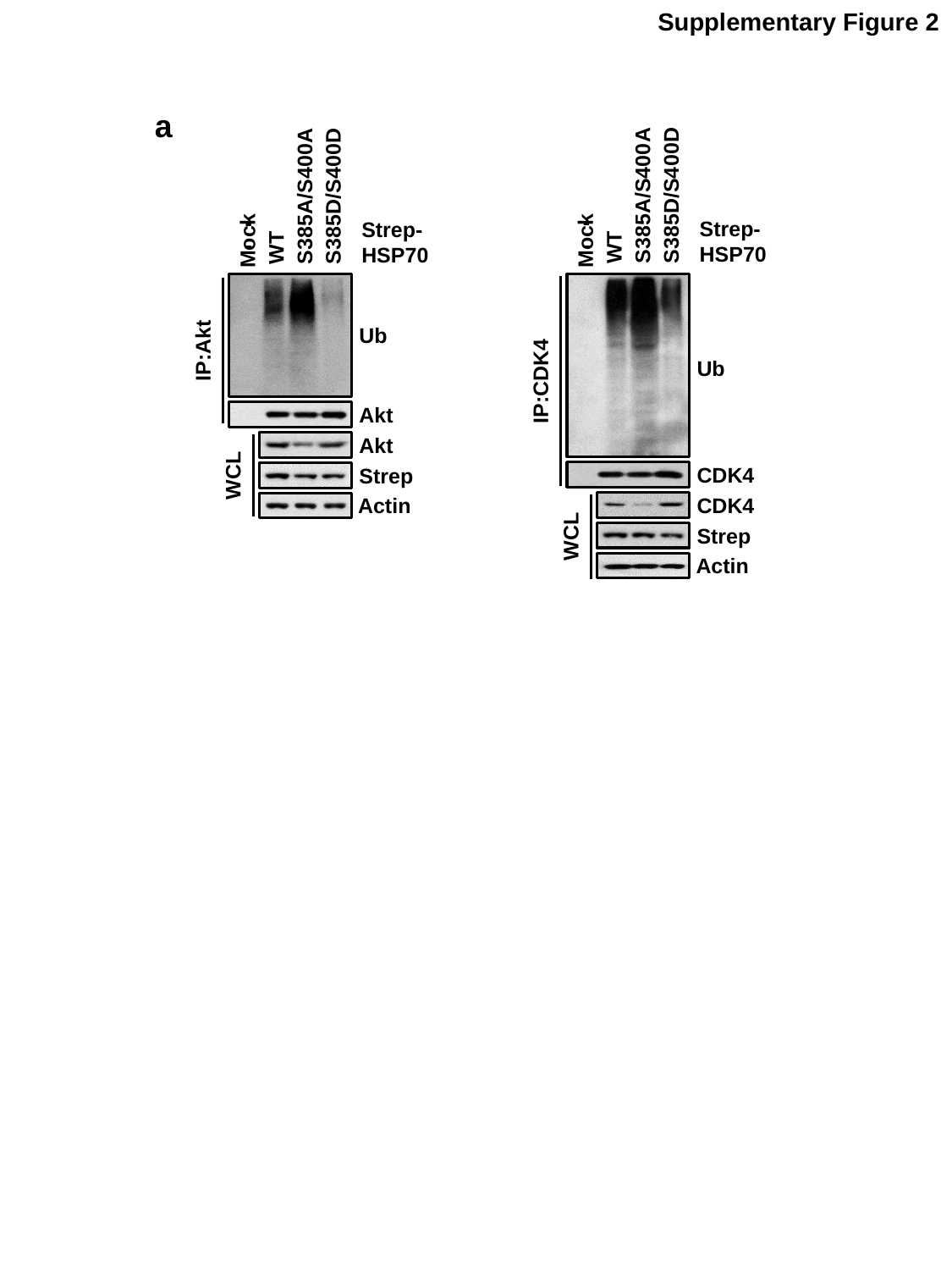

Supplementary Figure 2
S385A/S400A
S385D/S400D
-
Strep-
HSP70
WT
Mock
-
Ub
IP:CDK4
CDK4
CDK4
Strep
WCL
Actin
S385A/S400A
S385D/S400D
-
Strep-
HSP70
WT
Mock
-
Ub
IP:Akt
Akt
Akt
WCL
Strep
Actin
a

## Slide 3
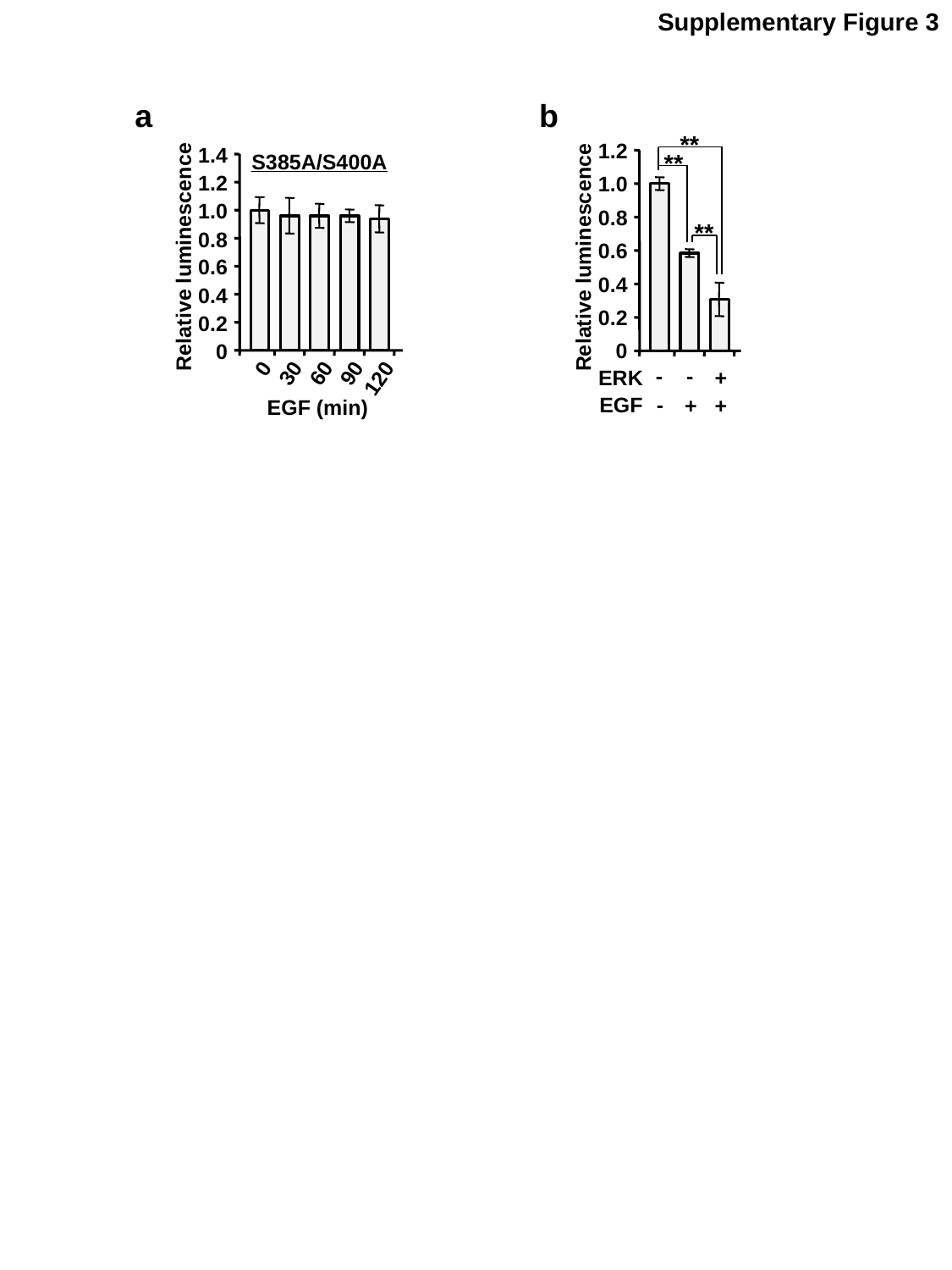

Supplementary Figure 3
a
b
**
1.2
**
1.0
0.8
**
0.6
Relative luminescence
0.4
0.2
0
-
-
ERK
+
-
EGF
+
+
1.4
S385A/S400A
1.2
1.0
0.8
Relative luminescence
0.6
0.4
0.2
0
EGF (min)
0
30
60
90
120

## Slide 4
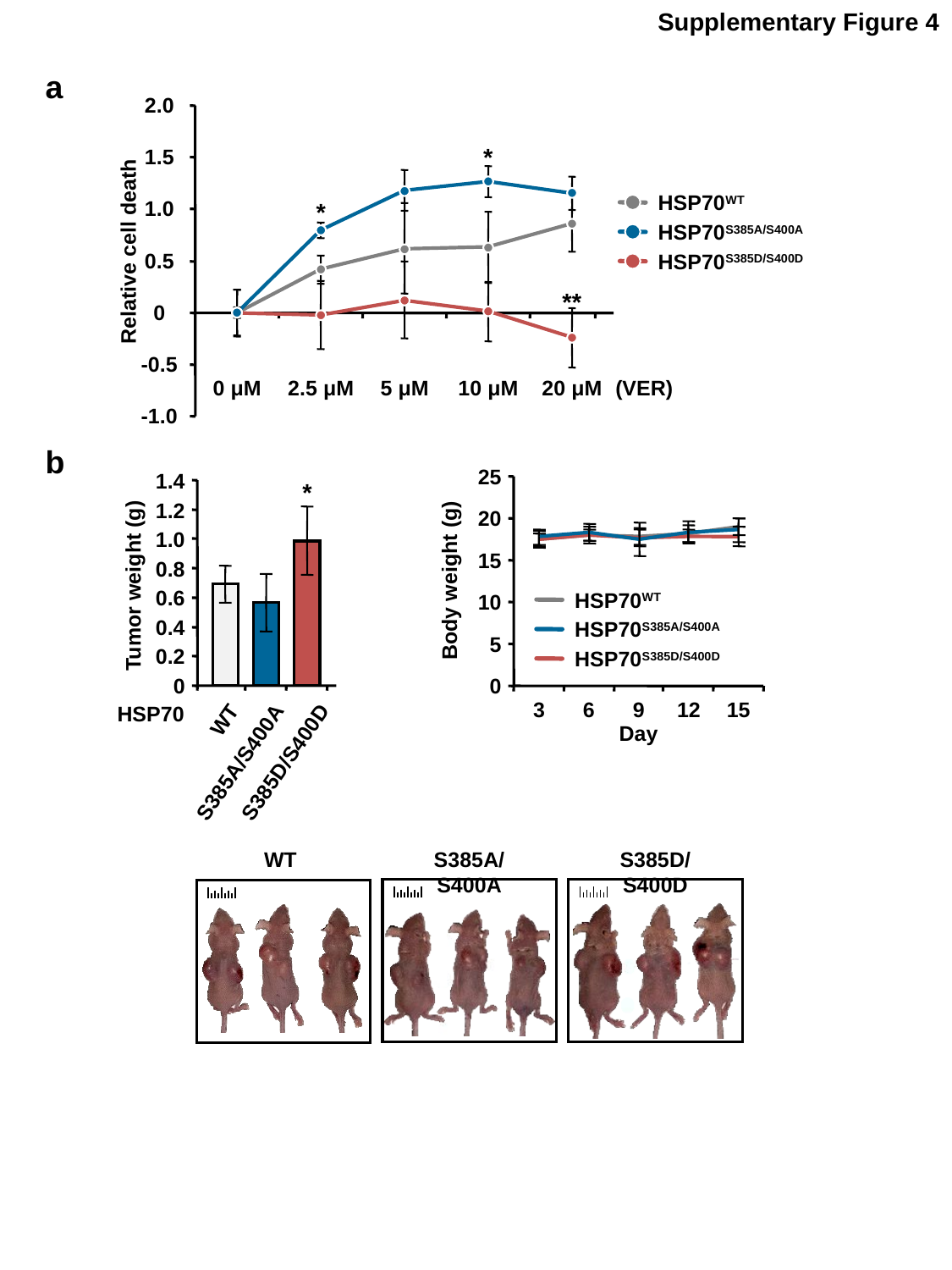

Supplementary Figure 4
a
2.0
*
1.5
HSP70WT
*
1.0
HSP70S385A/S400A
Relative cell death
HSP70S385D/S400D
0.5
**
0
-0.5
0 μM
2.5 μM
5 μM
10 μM
20 μM
(VER)
-1.0
b
25
20
15
10
5
0
Body weight (g)
3
6
9
12
15
Day
HSP70WT
HSP70S385A/S400A
HSP70S385D/S400D
1.4
*
1.2
1.0
0.8
Tumor weight (g)
0.6
0.4
0.2
0
HSP70
WT
S385A/S400A
S385D/S400D
WT
S385A/S400A
S385D/S400D
