# Supplementary material for: ERK-dependent phosphorylation of the linker and substrate-binding domain of HSP70 increases folding activity and cell proliferation
Source: Exp Mol Med. 2019 Sep 26;51(9):112. doi: 10.1038/s12276-019-0317-0 (PMC6802635; doi:10.1038/s12276-019-0317-0)
Supplement: Supplementary file 2 — Supplementary figure legends [file 12276_2019_317_MOESM2_ESM.docx]

**Supplementary information**

**ERK-dependent phosphorylation on linker and substrate-binding domain of HSP70 increases folding activity and cell proliferation**

Semi Lim^1^, Dae Gyu Kim^1^ and Sunghoon Kim^1,2^

^1^Medicinal Bioconvergence Research Center, College of Pharmacy, Seoul National University, Seoul, Korea. ^2^Department of Molecular Medicine and Biopharmaceutical Sciences, Graduate School of Convergence Science and Technology, College of Pharmacy, Seoul National University, Seoul, Korea.

Corresponding author: Sunghoon Kim (sungkim@snu.ac.kr)

Medicinal Bioconvergence Research Center, Department of Molecular Medicine and Biopharmaceutical Sciences, Graduate School of Convergence Science and Technology, College of Pharmacy, Seoul National University, Seoul, Korea.

Running title: Phosphorylation of HSP70 by ERK enhances activity

**Supplementary figure legends**

**Supplementary Fig. 1 EGF signal phosphorylates HSP70, not HSP90.** **a** Determination of phosphorylated HSP70 and HSP90 *via* various signaling pathway. 293T cells were treated with EGF, TNF-α and TGF-β signal, and subjected to immunoprecipitation with anti-HSP70 and -HSP90 antibody. Phosphorylation of HSP70 and HSP90 was monitored by SDS-PAGE and Western blotting using the indicated antibodies. Actin was used as a loading control. **b** Specific si-RNA targeting ERK was introduced into H460 cells, and the starved cells were treated with EGF and subjected to immunoprecipitation with anti-HSP70 antibody. The phosphorylation was monitored as above. **c** Starved 293T cells were transfected with Strep-HSP70 and GFP-ERK and treated with EGF for 10 minutes. The cells were subjected to immunoprecipitation. **d** *In vitro* binding between HSP70 and ERK. Purified GST-HSP70 were mixed with lysates expressing GFP-ERK and precipitated with glutathione-Sepharose beads. Co-precipitated ERK with HSP70 was detected by immunoblotting using anti-GFP antibody. GST proteins were observed by Coomassie staining.

**Supplementary Fig. 2 Phosphorylation of HSP70 affects the ubiquitination of client. a** Each of Strep-HSP70 wild type (WT), S385A/S400A and S385D/S400D was introduced into 293T cells. The cells were treated with MG-132 and subjected to immunoprecipitation using specific antibody against Akt (left) and CDK4 (right). Precipitates were separated by SDS-PAGE and ubiquitinated amounts of Akt and CDK4 were determined by immunoblotting with anti-Ub antibody. Actins was used as a loading control.

**Supplementary Fig. 3 Phosphorylation of HSP70 induces the structural change. a** The starved cells expressing HSP70 S385A/S400A tagged LgBiT and SmBiT at N- and C-terminal, respectively, (See Fig. 4e) were treated with EGF in time-dependent manner and luciferase signal was determined. Luciferase signals relative to un-treated sample were shown as bar graph. The experiments were independently repeated three times with error bars denoting S.D. **b** Significance of ERK and EGF signal for phosphorylation-dependent structural change of HSP70. The cells expressing of HSP70 conjugated LgBiT and SmBit at the N- and C-terminal, respectively, were overexpressed and stimulated with ERK and EGF, respectively, as indicated manner. Luciferase signal was determined as above and results relative to sample without ERK expression and EGF treatment was shown as a graph. Statistical analysis was performed with Student’s two-tailed t-test (**P*<0.05, ***P*<0.01).

**Supplementary Fig. 4 Phosphorylation of HSP70 enhances the cell proliferation. a** Each of 293T cells expressing HSP70 wild type (WT), S385A/S400A and S385D/S400D mutants were treated with different concentration of VER155008 (VER) and subjected to cytotoxicity assay. All the experiments were independently repeated three times with error bars denoting S.D. Statistical analysis was performed with Student’s two-tailed t-test (**P*<0.05, ***P*<0.01). **b** H460 cells stably expressing GFP-HSP70 wild type (WT), S385A/S400A or S385D/S400D were subcutaneously injected into the backs of BALB/cSLC-*nu/nu* mice. The tumor volume (Fig. 5c, right) and body weight (right) were monitored for the experimental period. Tumors were excised and weighed after sacrifice of the mice (left). Images of tumor-bearing mice were shown in below. Statistical analysis was performed with Student’s two-tailed t-test (**P*<0.05, ***P*<0.01).
